# Recurrent Acute Limb Ischaemia due to Malignant Thromboembolism Involving Bilateral Lower Limbs Simultaneously

**DOI:** 10.1002/cnr2.70591

**Published:** 2026-06-05

**Authors:** Amos Au, Audrey Choy, Ming Yii

**Affiliations:** ^1^ Monash Health Melbourne Victoria Australia; ^2^ School of Clinical Sciences Monash University Melbourne Australia

## Abstract

**Background:**

Acute Limb Ischaemia (ALI) is a limb and life‐threatening condition that is often associated with cardiovascular disease, atrial fibrillation (AF), diabetes and smoking. It is less commonly associated with active malignancy. There is growing evidence regarding this association. This case report details the case of a patient with active malignancy who had two separate episodes of ALI secondary to tumour thromboembolism, including both his lower limbs simultaneously.

**Case:**

A 68‐year‐old Caucasian male with a known history of Squamous Cell Lung Cancer on immunotherapy presented with ALI of his left upper limb that was managed surgically with a brachial embolectomy. Despite ongoing therapeutic anticoagulation, he presented again with ALI, this time involving both his lower limbs. This was treated successfully surgically, and the patient was discharged home after a prolonged period of rehabilitation. During this inpatient stay, he was found to have new cerebral metastases.

**Conclusion:**

This case highlights that oncological patients can have ALI that is not due to atherosclerotic disease alone and can still occur while patients are therapeutically anticoagulated. Furthermore, this case underscores the need for early involvement of oncology given that such presentations may indicate disease progression and indicate a poorer prognosis for many of such patients.

## 1 Introduction

Acute Lower limb Ischaemia (ALI) is a life‐threatening condition [1, 2, 3] that is often associated with cardiovascular disease, atrial fibrillation (AF), diabetes and smoking [3]. It is less commonly associated with malignancy [4, 5]. ALI usually affects, but is not limited to, the peripheral arteries, though it has been documented in both the visceral arterial supply and the venous system as well [4]. When ALI is missed or there are delays to treatment, it can lead to significant morbidity or patient mortality [1, 3]. Treatment for ALI can include open and/or endovascular techniques but will almost always include therapeutic anticoagulation [1, 3].

Malignancy has been rarely reported in the literature as the cause for ALI. Malignancy is understood to promote a hypercoagulable state, thus predisposing such patients to arterial and venous thromboembolic events [6, 7]. In addition to this, there is growing evidence to suggest that certain classes of immunotherapy such as PD–1 Inhibitors can induce arterial thrombosis by accelerating atherosclerotic plaque and localised inflammation [8].

Current evidence has predominately reported patients that have single limb involvement. No other cases were found in literature review which involve both recurrence and multiple limb involvement. In this unique case report, a rare and unfortunate case of recurrent ALI, including an episode of bilateral ALI of the lower limbs, secondary to malignant tumour thromboemboli, is presented.

## 2 Case

### 2.1 Initial Presentation

The patient, a 68‐year‐old Caucasian male, initially presented to Monash Medical Centre in December 2021 with sudden onset left hand pain. This was associated with discoloured fingers. The patient did not report any loss of power or paresthesia. There was no history of preceding trauma or surgery. The patient did not report being ill recently. The patient's past medical history included ischaemic heart disease (IHD) requiring coronary artery bypass grafting more than 10 years prior to his presentation. He also had System Lupus Erythematosus (SLE) for which he was on prednisolone and hydroxychloroquine at the time of presentation. Of note, he was an ex‐smoker at the time of presentation, with a 50‐pack year history. The patient did not report any upper or lower limb claudication or rest pain prior to his initial presentation. His significant oncological history is outlined below. His laboratory results on admission showed leucocytosis and neutrophilia and mildly elevated fibrinogen. The rest of his results were unremarkable (Table 1).

**TABLE 1:** Lab investigations.^a^

Test	First presentation	Second presentation
Hb	132 g/L (125–175)	**108** g/L L* (125–175)
WCC	**15.9** × 10^9^/L *H (4.0–11.0)	**15.9** × 10^9^/L *H (4.0–11.0)
Platelets	335 × 10^9^/L (150–450)	310 × 10^9^/L (150–450)
Neutrophils	(92.0%) **14.58** × 10^9^/L *H (2.00–8.00)	91.6% **14.56** × 10^9^/L *H (2.00–8.00)
Lymphocytes	(4.3%) **0.68** × 10^9^/L L* (1.00–4.00)	5.0% **0.79** × 10^9^/L L* (1.00–4.00)
Monocytes	(3.2%) 0.51 × 10^9^/L (0.20–1.00)	3.1% 0.49 × 10^9^/L (0.20–1.00)
Eosinophils	(0.2%) 0.03 × 10^9^/L (0.00–0.50)	0.1% 0.02 × 10^9^/L (0.00–0.50)
Basophils	(0.4%) 0.06 × 10^9^/L (0.00–0.20)	0.3% 0.05 × 10^9^/L (0.00–0.20)
INR	1.1 ratio (0.8–1.2 s)	2.5 ratio (H)
APTT	27 s (22–32 s)	32 s (22–32 s)
Fibrinogen	**6.2 g/L (H)** (1.5–4.0)	**5.0 g/L (H)** (1.5–4.0)
D‐Dimer	**0.77 mg/L (H)** (0.00–0.20)	**1.34 mg/L (H)** (0.00–0.20)
Sodium	138 mmol/L (135–145)	136 mmol/L (135–145)
Potassium	4.6 mmol/L (3.5–5.2)	4.8 mmol/L (3.5–5.2)
Chloride	101 mmol/L (95–110)	104 mmol/L (95–110)
Bicarbonate	27 mmol/L (22–32)	20 mmol/L (22–32) **(L)**
Urea	7.2 mmol/L (2.8–7.2)	7.8 mmol/L (2.8–7.2) **(H)**
Creatinine	82 mcmol/L (60–110)	80 mcmol/L (60–110)
eGFR	83 mL/min **(L)** (> 90)	86 mL/min (L) (> 90) **(L)**
ALP	**138 U/L (H)** (30–110)	108 U/L (30–110)
GGT	**62 U/L (H)** (5–50)	**54 U/L (H)** (5–50)
ALT	12 U/L (5–40)	8 U/L (5–40)
Total bilirubin	8 mcmol/L (0–20)	5 mcmol/L (0–20)
Albumin	36 (32–47)	37 (32–47)
Calcium	2.49 mmol/L (2.10–2.60)	2.02 mmol/L (2.10–2.60)
Corrected calcium	2.57 mmol/L (2.10–2.60)	2.20 mmol/L (2.10–2.60)
LDH	186 U/L (120–250)	Not done
High sensitive troponin	10 ng/L (0–20)	Not done

^a^ Normal ranges in parentheses, abnormal results in bold.

**FIGURE 1:**
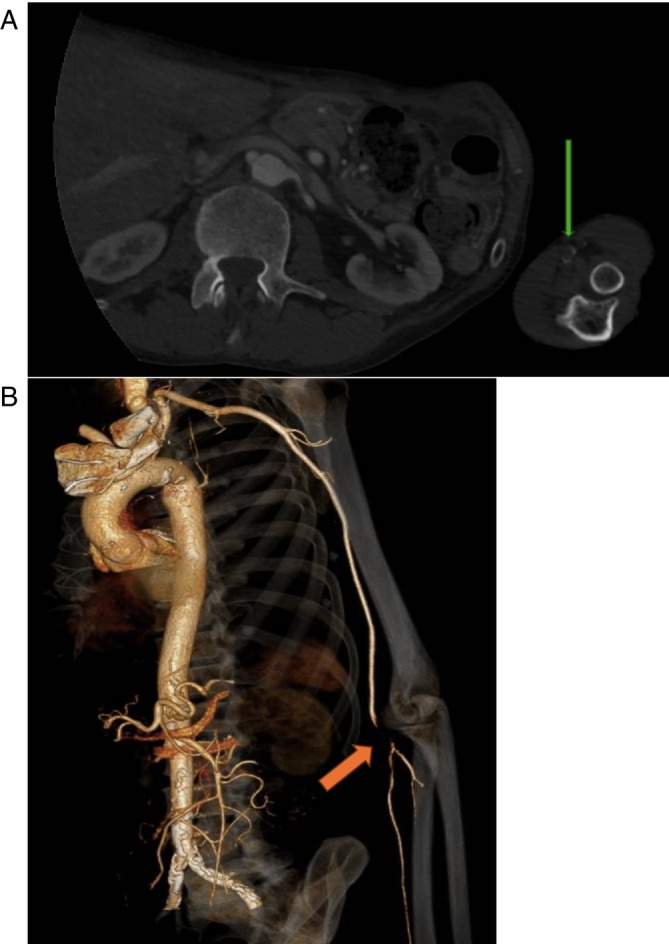
(A) CT Angiography (Arterial Phase) Showing Arterial Occlusion (Axial Slice). The green arrow shows a lack of contrast in distal brachial artery highlighting an occlusion in the artery. This is more easily appreciated in (B). This is a 3D Reconstruction of CT‐A of Upper Limb prior to the patient's first embolectomy. Please note the orange arrow highlighting the absence of continuity from the distal brachial artery to its bifurcation as it becomes the radial and ulnar arteries. This lack of continuity shows the absence of blood flow in the affected area.

**FIGURE 2:**
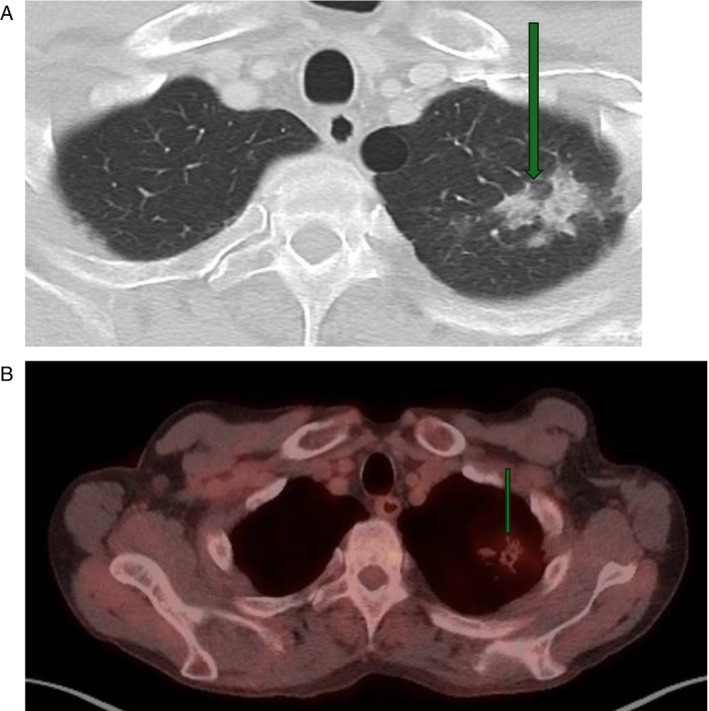
Previous CT chest showing patients malignancy (green arrow).

**FIGURE 3:**
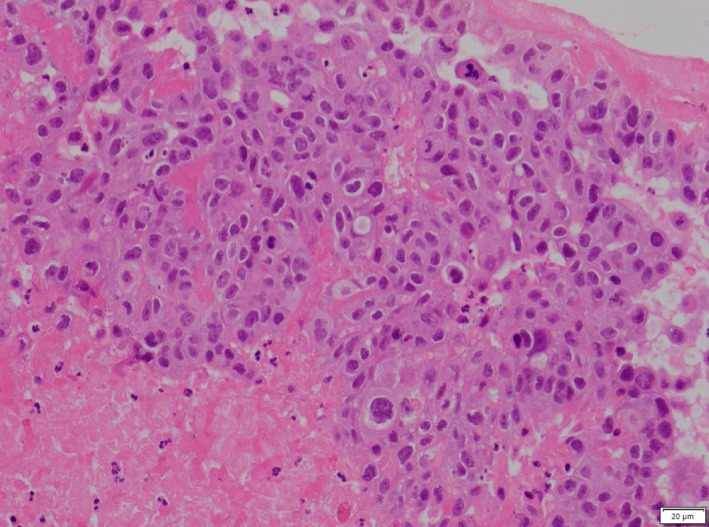
H&E upper limb thrombus (×200 magnification. Haematoxylin and Eosin staining).

CT Angiography (CT‐A) showed left brachial artery occlusion extending to the proximal radial and ulnar arteries (Figure 1A,B). Reconstituted flow was seen in the distal radial artery only. The patient was then started on a heparin infusion and then taken to theatre where an embolectomy was performed. Significant thromboembolic appearing material was retrieved from both radial and ulnar arteries and sent for histological analysis. At the end of the case, the hand was pink and warm with a strong radial pulse. The patient was well post operatively and on discharge his hand was pink and warm with no neurological deficits. Prior to discharge histology confirmed that the source of the occlusion was tumour embolus, consistent with his previous histological diagnoses of Squamous Cell Lung Cancer (SCLC) (Figures 2, 3). After discussion with the oncology and haematology teams, the patient was then discharged on warfarin. A subsequent post‐operative ultrasound 6 weeks later showed no residual thrombus in his brachial, radial and ulnar arteries.

### 2.2 Oncological History

The patient at the time of his first presentation was on Pembrolizumab as maintenance immunotherapy for metastatic SCLC, T4N2M0. This cancer was diagnosed 3 years prior following investigation into his recurrent laryngeal nerve palsy. Of note, his malignancy was histologically confirmed as SCC p40+, TTF‐1 Negative with no PDL1 He underwent chemoradiotherapy with a curative intent. He underwent chemoradiotherapy with curative intent. His chemotherapy regime was six cycles of carboplatin and paclitaxel and radiotherapy at 60GY at 30 fractions. Despite initial encouraging results, the patient had a recurrence of his malignancy a year later, which was confirmed radiologically and on histology. The patient was then started on Pembrolizumab. At the time of his first presentation the patient had already completed 18 cycles of Pembrolizumab (200 mg every 3 weeks). It should be noted that the decision to start the patient on Pembrolizumab was due to the patient's SLE and was unable to take durvalumab as originally intended. Please refer to Table 2 for a summarised timeline of the patient's clinical course.

**TABLE 2:** Timeline of patient progression.

August 2019	Diagnosis of squamous cell lung cancer (SCLC) following
October 2019	Chemoradiotherapy (carboplatin and paclitaxel) completed
August 2020	Relapse on PET – Confirmed on cytology of left supraclavicular node.
October 2020	Pembrolizumab started – nil issues
December 2021	Left Upper Limb Acute Limb Ischaemia (brachial artery occlusion) requiring surgical embolectomy. Started on warfarin on discharge. Histology consistent with known SCLC. Post‐operative duplex ultrasounds confirmed the absence of further thrombus.
August 2022	Bilateral Acute Lower Limb Ischemia (bilateral popliteal artery occlusion). Bilateral lower limb embolectomies and fasciotomies performed. During this admission, cerebral metastases were discovered.
January 2023	Whole brain radiotherapy
March 2023	Patient deceased

### 2.3 Second Presentation

**FIGURE 4:**
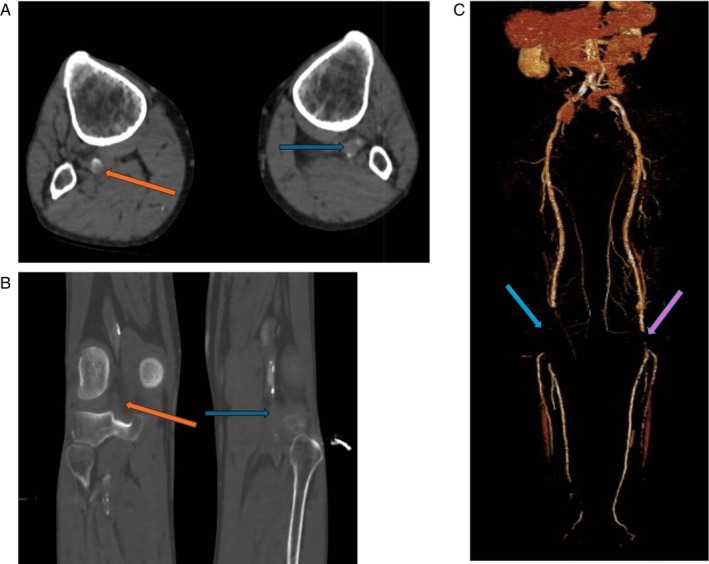
(A) CT‐A with lower limb runoff (further distal) showing occlusion of the right (orange arrow) as well as the left (blue arrow) popliteal artery. This is evident in the lack of contrast in the popliteal arteries shows that there is an occlusion at the level of the popliteal arteries bilaterally. (B) Saggital Views of Popliteal Artery Occlusions: Similarly to the axial views, there is a lack of contrast in the popliteal arteries in this view. (C) 3D Reconstruction of CT‐A of Lower Limbs prior to the patient's second operation. Please note both arrows highlighting the absence of continuity in this 3D reconstruction. On the patient's Right (blue arrow), this is from the origin of the popliteal artery to the origins of the anterior tibial artery and the tibioperoneal trunk. On the left (purple arrow) the second part of the popliteal artery is missing. The lack of continuity in the lower limb vasculature shows the absence of blood flow in the affected areas signifying occlusion.

In August 2022, 8 months after his presentation for ALI of his left arm, the patient presented with sudden onset bilateral lower limb pain to the same institution. Like his previous presentation, there was no clear precipitant, no preceding claudication or rest pain‐like symptoms. The patient only experienced sudden onset pain and discolouration in both his lower limbs. On this presentation, there were femoral pulses palpated bilaterally only, and his left foot on examination was insensate with minimal movement of his foot, consistent with a Rutherford 2B Score (for acute limb ischaemia) [9]. The patient's right foot was not as severe, with sensory deficits in the foot noted but with his motor function preserved; this was consistent with a Rutherford 2A Score. Both of his lower limb compartments were soft; however, the left was more painful than the right on exam, especially on passive movement. CT A confirmed that he had occlusion of his left popliteal artery, with scant opacification of his tibial arteries distal to this (Figure 4A–C). Similarly, on the other side, his right popliteal artery was also occluded, with very little contrast observed in his tibial arteries (Figure Error! Reference source not found.).

ECG showed no signs of AF and the patient and his family confirmed that he had been compliant with his warfarin therapy. His INR was 2.5 (normal range 0.8–1.2) on presentation, which was within the therapeutic target of 2.0–3.0 given his previous presentation for limb ischaemia. Additional baseline blood tests were also obtained and were similar to his previous presentation, showing a leucocytosis and neutrophilia but no other significant biochemical derangement (Table 1).

The patient was commenced on a heparin infusion and taken to the operating theatre where bilateral lower limb embolectomies were performed (Left then Right). Similarly, like his first operation, embolic material resembling tumour thrombus was removed with a three French Fogarty catheter. This was done via a popliteal approach, where the below knee popliteal artery was exposed, and embolic material was retrieved from his tibial vessels. There was no significant calcification noted at the popliteal vessels on entry, or any stenoses when trawling the femoral and tibial arteries of both legs. Prior to finishing the operation, there was good inflow and backflow into the popliteal artery in both legs. Given concerns about the firmness of the left lower limb at the end of the case, a full four‐compartment fasciotomy was performed. On examination of the muscles of each of the four compartments, all were of appropriate colour and responding to diathermy. His feet were pink and warm at the end of the case.

**FIGURE 5:**
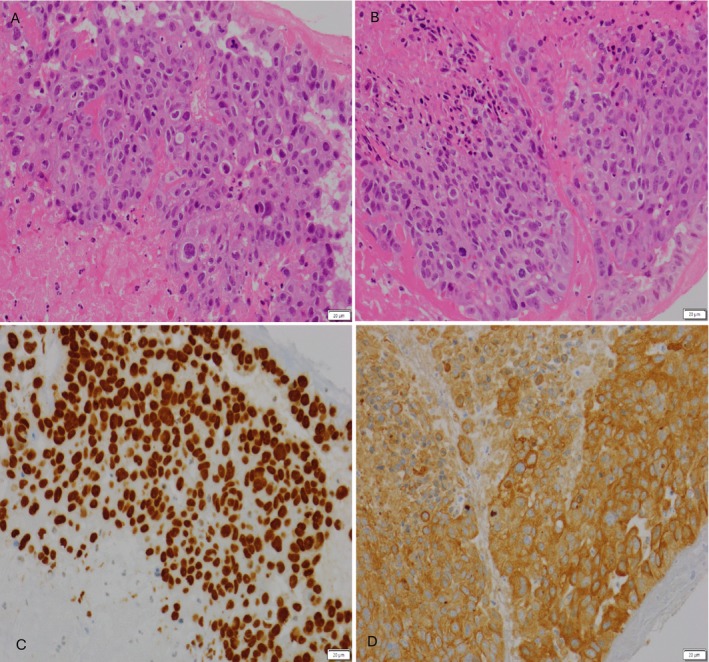
(Clockwise) (A) (H&E, ×200 magnification) and (B) (H&E, x200 magnification) show the upper limb and lower limb thrombus on Haematoxylin and Eosin staining. At high power, the tumour cells have pleomorphic, hyperchromatic nuclei with nucleoli, scattered mitotic figures and dense eosinophilic cytoplasm. Focal dyskeratotic cells are seen. The tumour nests are surrounded by fibrin and haemorrhage. (C) Shows positive staining for P40 in the tumour cells, and (D) shows positive staining for AE1/AE3 in the tumour cells, in keeping with metastatic squamous cell carcinoma (×200 magnification).

Like his previous operation, the embolic material was sent to our pathology department for histological analysis. This confirmed that the embolus was malignant in nature and consistent with his known SCLC (Figure 5).

**FIGURE 6:**
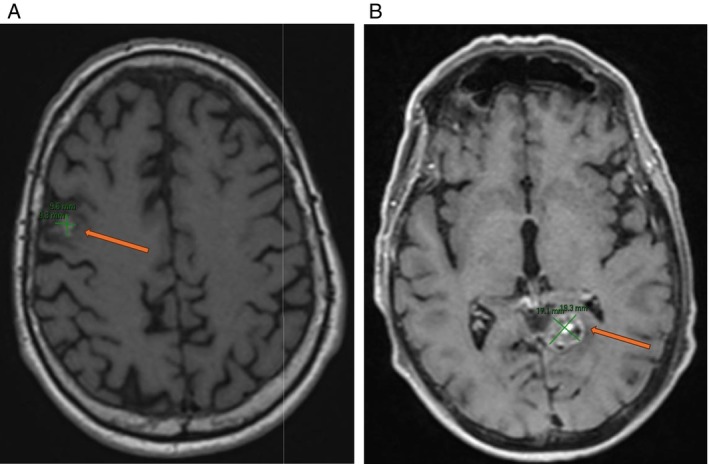
(A) MRI‐B. 10 × 9 mm lesion in the right middle frontal gyrus centred at the grey‐white matter junction. The lesion demonstrates intrinsic T1 hyperintensity with no appreciable enhancement. There is surrounding T2/FLAIR signal hyperintensity involving the subcortical white matter and cortex. There is extensive SWI signal abnormality corresponding to calcification on prior CT. (B) MRI‐B. 19 mm medial left occipital lobe metastasis with mild vasogenic oedema.

The patient recovered gradually and required split skin grafts to help close his fasciotomy wounds. Unfortunately, the patient experienced new seizures prior to discharge, and given his oncological history, an MRI‐Brain was performed which showed new cerebral metastases (Figure 6A,B). The patient continued to recover from the surgery; his feet remained pink and warm, though histology from the operation confirmed that this presentation was again due to tumour emboli. After his inpatient care, the patient was transitioned to the community for ongoing rehabilitation, where he followed up in the outpatient clinic. Post‐operative ultrasound showed patent lower limb vasculature. In addition to this, a transthoracic echocardiogram (TTE) did not show a PFO or any large thrombus in his heart. During this time, the patient underwent whole brain radiotherapy for symptom management of his cerebral metastases.

Unfortunately, 7 months after his presentation for bilateral lower limb ischaemia, and the discovery of new cerebral metastases, the patient passed away due to advanced disease after exhausting all treatment options.

## 3 Discussion

This is a case report that describes a case of tumour emboli causing ALI in the upper and bilateral lower limbs on two distinct presentations. Currently there is paucity in the literature on tumour emboli causing ALI. There has been a systematic review of this topic, but it is limited only to observational data with a heavy weighting on a single study [10]. It should be noted that this review did not include a breakdown of which limbs were affected or if recurrent ALI was observed [10]. A European multi‐centre (retrospective) study assessing treatment efficacy of 139 patients over a 12 year period studying ALI in patients with active neoplastic disease only included patients with lower limb ALI without any histological analysis of the tumour thrombus [11]. Prior to these studies, there have been several case reports published as well as a limited review attempting to summarise these cases [4, 6, 12, 13].

Cancer is a known hypercoagulable state [6, 10], and tumour emboli have been shown to cause ALI as well as other ischaemic phenomena including pulmonary embolism and ischaemic bowel [4, 6, 12, 13]. In the systematic review by Hussain, the most common malignancies were non‐small cell lung cancer and atrial myxoma [4]. In a separate review, Govsyeyev et al. found that nearly three quarters of patients with ALI had either a cancer of the skin and soft tissue (19%), genitourinary (18%), lung (17%), and gastrointestinal (16%) systems [10] which is in keeping with the limited literature regarding this phenomenon [4, 5, 6, 13, 14]. The management of this patient is consistent with other cases described in the literature; initiation of a therapeutic heparin infusion followed by urgent surgery to minimise morbidity. Given the rare presentation of ALI secondary to tumour embolus in patients, there is no guidelines as to suggest best practice. A single centre, retrospective analysis of 74 patients with concurrent malignancy and ALI concluded that there was no difference in their centre when comparing open surgery (i.e., thrombectomy) and an endovascular approach (i.e., catheter directed lysis) [12], which is consistent with current evidence regarding the management of ALI of non‐malignant origins [3].

There is currently a lack of evidence regarding the role and efficacy of therapeutic anticoagulation as an isolated strategy in the management of arterial tumour thrombus. In the case of arterial ischaemia, surgical management is understood to be best practice in most cases [4, 12, 14], and as such there is a paucity of cases that receive only medical management [15], which makes it difficult to assess the efficacy of anticoagulation in isolation from other treatments. Additionally, the bulk of current research focuses on the more common venous thromboembolism [15, 16] with little emphasis on arterial tumour thrombus.

The patient described in this case report lived to experience another episode of ALI despite being appropriately anticoagulated; no similar cases have been identified of any other recurrent episodes of tumour embolisation causing ALI. The patients described in a summary review of previous cases of arterial tumour emboli appear to have either been discharged or not survived their presentation. It remains unclear if these surviving patients lived long enough to have recurrent episodes of arterial tumour embolisation.

In the literature, the presence of tumour embolisation appears to be an indicator of disease progression [5, 13]; as was the case for this, patient who was diagnosed with cerebral metastases shortly after second operation. Two studies with a small number of cases of ALI with patients who had active malignancy have advocated for strong consideration of comfort care given the poor outcomes of their patients [5, 13]. Another paper has argued against this based on their single centre experience over a 10‐year period, citing an 80% 1 year survival rate [12]; however, only 43% of the patients in the same study avoided a major limb amputation [12].

This is not the first case of thrombosis of a patient on Pembrolizumab [8, 17]. There has been a growing amount of evidence linking immune checkpoint inhibitors such as pembrolizumab to increased thrombotic events due to the action of the PD‐1 blockade, which may accelerate the development of atherosclerotic plaque [8]. It is unclear the role immunotherapy has played in the development of this patient's clinical picture due to several factors. While his previous significant history of AMI and smoking does indicate some degree of atherosclerotic change, this was not evident on thorough macroscopic examination of peripheral vessels during his operations, and as such was not felt to be a significant contribution to these presentations. Additionally, the patient's limbs had not been imaged previously, preventing any comparisons. Furthermore, the operating team did not take samples of the patient's vessel for histological analysis to check for atherosclerotic changes given the operative findings encountered. Histological analysis demonstrated that the embolic material on both occasions was malignant in origin; however, a degree of underlying contribution from atherosclerosis cannot be excluded, and as such the degree of contribution of immunotherapy to this presentation is unclear. The patient that was described in this case report had his malignancy managed by the same hospital network as the Vascular Surgery team, making it easier to confirm his history of malignancy. It is imperative in the setting of ALI, especially in patients that do not appear to have an active malignancy (or a previous history), to send histology of any thrombus retrieved when performing the arterial embolectomy. This not only confirms the presence of tumour emboli, but it can also confirm the presence of a new malignancy [14].

## 4 Conclusion

ALI due to tumour thromboembolism is a rare occurrence. It should never be overlooked as an underlying cause, especially when the primary tumour is still in situ. Furthermore, there is growing evidence of the possible association between immune checkpoint inhibitors and thrombotic events. It is important to recognise that ALI can manifest elsewhere after the initial episode despite appropriate anticoagulation management in between episodes and that it may not be isolated to a single limb. When counselling patients with ALI due to tumour emboli, it is important to involve the oncology teams and to note that such a presentation may indicate further cancer progression.

## Author Contributions

**Ming Yii:** conceptualization, supervision, writing – review and editing. **Amos Au:** conceptualization, investigation, writing – original draft, methodology, writing – review and editing. **Audrey Choy:** investigation, visualization, data curation.

## Funding

The authors have nothing to report.

## Conflicts of Interest

The authors declare no conflicts of interest.

## Data Availability

The data that support the findings of this study are available from the corresponding author upon reasonable request.
